# Micronutrients May Be a Unique Weapon Against the Neurotoxic Triad of Excitotoxicity, Oxidative Stress and Neuroinflammation: A Perspective

**DOI:** 10.3389/fnins.2021.726457

**Published:** 2021-09-22

**Authors:** Kathleen F. Holton

**Affiliations:** Nutritional Neuroscience Lab, Department of Health Studies, Center for Neuroscience and Behavior, American University, Washington, DC, United States

**Keywords:** excitotoxicity, antioxidants, oxidative stress, neuroinflammation, neurotoxic triad, vitamins, glutathione

## Abstract

Excitotoxicity has been implicated in many neurological disorders and is a leading cause of oxidative stress and neuroinflammation in the nervous system. Most of the research to date has focused on each of these conditions individually; however, excitotoxicity, oxidative stress, and neuroinflammation have the ability to influence one another in a self-sustaining manner, thus functioning as a “neurotoxic triad.” This perspective article re-introduces the concept of the neurotoxic triad and reviews how specific dietary micronutrients have been shown to protect against not only oxidative stress, but also excitotoxicity and neuroinflammation. Future dietary interventions for neurological disorders could focus on the effects on all three aspects of the neurotoxic triad.

## Introduction

**FIGURE 1:**
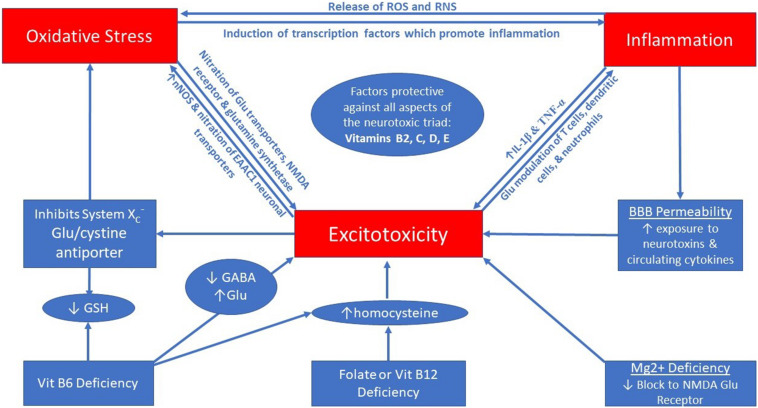
The neurotoxic triad is a self-perpetuating cycle between excitotoxicity, oxidative stress, and neuroinflammation.

Excitotoxicity has been implicated in many neurological disorders including, but not limited to, Alzheimer’s (Wang and Reddy, 2017), Parkinson’s (Iovino et al., 2020), amyotrophic lateral sclerosis (ALS; King et al., 2016), multiple sclerosis (MS; Kostic et al., 2013), epilepsy (Vishnoi et al., 2016), migraine (Longoni and Ferrarese, 2006), chronic pain (Inquimbert et al., 2018), and psychiatric conditions (Olloquequi et al., 2018). Excitotoxicity is characterized by over-excitation of neurons which leads to neuronal cell death, and this process is primarily mediated by high amounts of the excitatory neurotransmitter glutamate (Olney, 1990). This process potentiates oxidative stress in the nervous system (Bondy and LeBel, 1993) and the interplay between excitotoxicity, oxidative stress, and neuroinflammation is thought to be associated with neurodegenerative conditions; thus, being an important target for future therapies (Chamorro et al., 2016; Kamal et al., 2020). These three biological states appear to be able to influence one another in a self-sustaining cycle (Nguyen et al., 2011) forming a “neurotoxic triad.” Figure 1 illustrates the self-sustaining interaction between excitotoxicity, oxidative stress, and inflammation (which are reviewed below), while also presenting some nutritional deficiencies which can fuel the triad.

The objective of this perspective article is to re-introduce the concept of the neurotoxic triad with a focus on the self-sustaining interaction between excitotoxicity, oxidative stress, and neuroinflammation; and to examine why dietary micronutrients could play a central role in the treatment of disorders characterized by the neurotoxic triad.

## Current Status of Knowledge

Most of the research to date has evaluated each aspect of the neurotoxic triad individually, in regards to one or more neurological/psychiatric conditions, or has focused on the intersection of only two components. A few authors have written about the co-occurrence of excitotoxicity, oxidative stress, and neuroinflammation (Chamorro et al., 2016; Ambrogini et al., 2019; Kamal et al., 2020), but to date, little attention has been paid to the reinforcing properties of these three detrimental states. Nguyen et al. (2011) have illustrated the cyclical properties of excitotoxicity, oxidative stress, and changes in mitochondrial function in mice, but this work did not evaluate inflammation. The novel perspective presented herein aims to bridge the current knowledge base to demonstrate the intricate and reinforcing connection between excitotoxicity, oxidative stress, and neuroinflammation while presenting an example of how specific micronutrients may have the unique ability to protect against all three aspects of the neurotoxic triad.

## Neurotoxic Triad – Excitotoxicity, Oxidative Stress and Neuroinflammation

Excitotoxicity occurs when abnormally high levels of glutamate are in the synaptic cleft between neurons, leading to prolonged activation of glutamate receptors, such as the NMDA receptor, resulting in increased calcium influx into the postsynaptic neuron and resultant cell death (Olney, 1990). Glutamate excitotoxicity can lead to increased production of neuronal nitrogen oxide synthase (nNOS) which in turn can lead to higher production of reactive nitrogen species, damage to cellular components, and ultimately, cell death (Chen et al., 2017). Excitotoxicity can impair mitochondrial function, leading to altered cellular energy production (Sullivan and Brown, 2005), and this is thought to be mediated by the oxidative stress triggered by excitotoxicity (Nguyen et al., 2011). Additionally, mitochondrial dysfunction has been associated with increased expression of NMDA receptors (Nguyen et al., 2011). An excellent review of this interaction has been published please see Gillessen et al. (2002). Glutamate release can also directly affect inflammation through its ability to stimulate immune cells. For example, T cells are well known for their ability to respond to glutamate concentration, as they contain both ionotropic and metabotropic glutamate receptors (Ganor and Levite, 2014). In disorders such as multiple sclerosis, glutamate is thought to stimulate production of autoreactive T cells (Ganor and Levite, 2014), which may be due to stimulation of AMPA glutamate receptors on the cell surface (Ganor et al., 2007).

Oxidative stress also has the ability to directly affect excitotoxicity and neuroinflammation. An excellent example of this is the action of reactive nitrogen species (RNS) on glutamate transporters. Glutamate transporters are necessary for clearing excess glutamate from the synaptic cleft to prevent excitotoxicity (Martinez-Lozada et al., 2016; Rose et al., 2018). Some glutamate transporters also facilitate the uptake of cysteine, which is the rate limiting factor in the production of the endogenous antioxidant glutathione (Lu, 2009). Nitration of glutamate transporters can inhibit their function, increasing the likelihood of excitotoxicity (Chen et al., 2010). Similarly, nitration of glutamine synthetase (GS) can also increase neuronal excitation via decreased conversion of excitatory glutamate to non-toxic glutamine before transport between astrocytes and neurons (Muscoli et al., 2013). Additionally, this can cause astrocytic swelling, which leads to channel opening that has been associated with astrocytic release of glutamate, which would further support excitotoxicity (Kimelberg et al., 1990). Peroxynitrite (combination of a superoxide radical with nitric oxide) has the ability to interact with NMDA glutamate receptors, leading to the nitration of NR1 receptor subunits (Lafon-Cazal et al., 1993). This change supports activation of NMDARs ultimately leading to excitotoxicity (Mishra and Delivoria-Papadopoulos, 1999; Zanelli et al., 2000). Moreover, peroxynitrite has been implicated in the nitration of the EAAC1 neuronal transporters, reducing the cell’s ability to take up cysteine and inhibiting the intracellular neuronal production of the endogenous antioxidant glutathione, which can also lead to cell death due to the inability to counter intracellular oxidative stress (Aoyama et al., 2008). Thus, peroxynitrite has the ability to further potentiate oxidative stress by reducing the rate-limiting substrate for the production of glutathione, in addition to being able to increase excitotoxicity, making it especially detrimental for the nervous system. Additionally, oxidative stress can also stimulate neuroinflammation directly (Bulua et al., 2011) and scavenging of ROS has been shown to effectively reduce neuroinflammation induced by seizures in an animal model (McElroy et al., 2017).

Not only can excitotoxicity and oxidative stress lead to neuroinflammation, but neuroinflammation caused by trauma, stress, or infiltration of peripheral cytokines, can also independently cause both excitotoxicity and oxidative stress (Hewett et al., 1994; Poletti et al., 2018). Microglia are the main immune cells of the nervous system, and these mediate neuroinflammation through the release of inflammatory cytokines such as IL-6, TNF-α, and IL-1β, in addition to chemokines and secondary messengers such as nitric oxide (NO; Becher et al., 2017). Two of these cytokines, TNF-α and IL-1β, have been shown to increase excitatory neurotransmission via glutamate (Kawasaki et al., 2008; Olmos and Llado, 2014), thereby allowing these two cytokines to influence excitotoxicity. Neuroinflammation can lead to the release of ROS as well as RNS, thereby independently increasing oxidative stress in the nervous system (Brown and Neher, 2010; Kempuraj et al., 2016). During pathological conditions, cytokines can also stimulate inducible nitrogen oxide synthase (iNOS) which results in the production of NO in astrocytes and microglial cells (Murphy, 2000), which can then combine with superoxide radicals to form the aforementioned peroxynitrite, facilitating excitotoxicity (Leist et al., 1997) and DNA damage, ultimately leading to cell death (Xia et al., 1996).

In summary, the neurotoxic triad is self-perpetuating, with each component able to support the propagation of the others. Thus, any disorder for which excitotoxicity has been implicated, will have a high likelihood of oxidative stress and neuroinflammation also being present. Optimal treatment options may need to address all three aspects of the neurotoxic triad, thereby stopping this self-perpetuating cycle. Interestingly, micronutrients may be a leading treatment contender due to their ability to protect against excitotoxicity, oxidative stress, and neuroinflammation concurrently. Below, we review micronutrients of particular interest due to their ability to protect against all three aspects of the neurotoxic triad, as well as a few micronutrients which can protect against two aspects of the triad, with potential indirect effects on the third. Effects of these micronutrients on each aspect of the neurotoxic triad, in addition to food sources, and some clinical findings are listed in Table 1.

**TABLE 1:** Micronutrients which protect against excitotoxicity, oxidative stress, and/or neuroinflammation.

	Protective action			Examples of clinical research	Food sources*
	Excitotoxicity	Oxidative stress	Neuroinflammation		
**Vitamin C**	-Attenuates activity of the NMDA receptor -Promotes clearance of Glu from synaptic cleft -Inhibits calcium channels	-Water soluble intracellular antioxidant -Maintains Vitamin E’s reducing capability	-Reduces microglial activation -Reduces production of the inflammatory cytokines IL-1β and TNF-α, which both increase glutamatergic neurotransmission	-Lower serum concentrations of Vitamin C in MS patients as compared to healthy controls (Besler et al., 2002) -Lymphocyte Vitamin C levels were lower in more severe, as compared to less severe Parkinson’s patients -Preoperative dosing of Vitamin C has been shown to reduce postoperative pain (Chen et al., 2016) -Lower plasma vitamin C in subjects with dementia as compared to healthy controls (Charlton et al., 2004) -Combined supplementation with Vitamin C and Vitamin E associated with reduced risk of Alzheimer’s (Zandi et al., 2004)	Goji berries, Bell peppers, Citrus fruit, Kiwis, Greens, Other fruits and vegetables
**Vitamin E**	-Increases activity of glutamine synthetase -Decreases activity of Glu receptors	-Antioxidant for tissues with high lipid content -Protects mitochondrial membrane potential	-Reduces neuroinflammatory responses by microglial cells -Lowered hippocampal levels of IL-1β and TNF-α in animal model of epilepsy	-Lower serum concentrations of Vitamin E in MS patients as compared to healthy controls (Besler et al., 2002) -Decreased seizure frequency, improved EEG, and reduced inflammatory markers in epilepsy patients (Mehvari et al., 2016) -Higher serum tocopherol associated with reduced risk of cognitive impairment in older adults (Mangialasche et al., 2013) -Higher intake of Vitamin E was found to be protective against Parkinson’s onset (Chang et al., 2021) -Combined supplementation with Vitamin C and Vitamin E associated with reduced risk of Alzheimer’s (Zandi et al., 2004)	Seeds, Nuts, and Avocado (and their respective oils), Bell peppers, Sweet potatoes, Green leafy vegetables, Fish
**Vitamin D3**	-Reduces excitotoxicity through modulation of NMDA receptors	-Along with vitamin A, regulates gene transcription for antioxidant enzyme systems	-Reduces microglial activation -Increases the anti-inflammatory cytokine IL-10	-Vitamin D deficiency associated with increased risk of MS -Schizophrenia patients have higher likelihood of vitamin D deficiency as compared to age-matched controls -Children with ASD had lower vitamin D levels at birth than their healthy siblings	Fish, Cod liver oil, Butter, Eggs, Meat, Milk (Vaes et al., 2017)
**Riboflavin (Vitamin B2)**	-Supports formation of active form of vitamin B6 (PLP) needed for conversion of Glu to GABA -Prevents formation of the NMDA agonist quinolinic acid in the kynurenine pathway -Helps prevent build-up of homocysteine	-Supports glutathione reductase -Deactivates lipid peroxides -Appears to have independent antioxidant activity	-Shown to reduce TNF-α, IL-1β, and nitric oxide -Supports vitamin D metabolism (including Vitamin D’s anti-inflammatory effects)	-Higher intake of riboflavin improves motor symptoms in MS patients (Naghashpour et al., 2017) -Has been shown to reduce migraine frequency in adults (Thompson and Saluja, 2017)	Eggs, Beef, Fish, Milk, Yogurt, Almonds, Avocado, Turkey, Millet, barley, buckwheat
**Vitamin B6**	-Essential cofactor for the conversion of Glu to GABA, thereby ↓excitotoxicity and ↑inhibition	-Indirect effects – Cofactor in the conversion of homocysteine to cysteine, allowing it to be used in the formation of glutathione	-Inhibits NLRP3 inflammasome activation, which decreases production of IL-1β	-Has been shown to reduce seizures in drug resistant epileptic patients (Ohtahara et al., 2011)	Potatoes, Pork, Chicken, Fish, Banana, Beef, Avocado
**Vitamin B12**	-Deficiency in B12 (and/or folate) can result in ↑homocysteine, which acts as a neurotoxic agonist of the NMDA receptor	-May indirectly affect antioxidant capacity through one carbon metabolism (along with folate)	-Deficiency associated with TNF-α induced neuroinflammation and higher systemic IL-6	-Deficiency associated with increased IL-6 production and altered Treg cells peripherally in Alzheimer’s patients (Politis et al., 2010) -B12 deficiency associated with Alzheimer’s, Parkinson’s and vascular dementia, but supplementation only appears to help those with B12 deficiency (Moore et al., 2012)	-Only found in animal products (Clams, Liver, Mussels, Fish, Oysters, Pork, Beef, Lamb, Milk, Eggs)
**Magnesium**	-Blocks NMDA receptor protecting against excitotoxicity	-Possible indirect effects, mechanism is debated	-Shown to inhibit L-type calcium channels and NF-κB signaling of microglia exposed to LPS -Reduced TNF-α and IL-1β, and improved short-term memory deficits in rats	-Neuroprotective for pre-term infants, protects against severe cerebral palsy (Rouse et al., 2008) -Reported benefit of improving stroke outcomes with IV Mg, but meta-analysis not significant -Lower Mg^2+^ levels in epilepsy patients as compared to controls -Mg^2+^ strongly associated with migraine (Dolati et al., 2020), has been shown to alleviate multiple types of pain, and reduce depression and anxiety (Tarleton et al., 2017)	-Seeds (especially pumpkin seeds), Nuts, Salmon, Buckwheat, Potatoes, Mackerel

The first four micronutrients have shown strong potential for protecting against all three components of the neurotoxic triad, while the others affect two aspects of the triad, with potential for indirect effects on the third aspect. *Foods highest in each micronutrient were identified using the USDA Human Nutrition Research Center food composition tables.

## Vitamins C and E

Two vitamins which are well known for their antioxidant capabilities, vitamin C and vitamin E, have been shown to also be protective against excitotoxicity and neuroinflammation. Vitamin C (ascorbic acid) is distributed throughout the body, but is found in highest concentration in the brain (Smythies, 1996). In the central nervous system, vitamin C acts as a cofactor in monoamine neurotransmitter production for dopamine and norepinephrine (May et al., 2012, 2013; Meredith and May, 2013), contributes to myelinization of axons (Olsen and Bunge, 1986**;** Eldridge et al., 1987), and to prenatal (Meredith and May, 2013) and postnatal (Pastor et al., 2013) brain development, in addition to its very important role as an intracellular antioxidant (Padayatty et al., 2003). Vitamin C plays a major antioxidant role in the aqueous parts of the cell and also helps maintain the reducing capability of vitamin E for protection of cell membranes (Beyer, 1994; May, 1999**;** Getoff, 2013). Additionally, vitamin C has been shown to be protective against glutamate induced excitotoxicity (Majewska et al., 1990; MacGregor et al., 1996; Matarredona et al., 1997). It can attenuate activity of the NMDA receptor (Majewska et al., 1990), enhance the uptake of glutamate from the synaptic cleft (Lane and Lawen, 2013), and directly inhibit calcium channels, thereby modulating neuronal excitability (Nelson et al., 2007). Additionally, reductions in neuroinflammation have been observed in animal models. Pretreatment with vitamin C has been shown to reduce microglial activation and production of the proinflammatory cytokines TNF-α and IL-1β in an LPS model in mice (Zhang et al., 2018). Similarly, vitamin C was shown to reduce microglial activation in a rat model of ethanol induced neuroinflammation (Ahmad et al., 2016). Additionally, double deficiency in vitamins C and E have been shown to cause increased neuroinflammation, while also impairing conditioned fear memory, in mice (Takahashi et al., 2019).

Similar to vitamin C, vitamin E also appears to affect every aspect of the neurotoxic triad (Ambrogini et al., 2019). Vitamin E is well known for its chain breaking antioxidant function where it has the ability to stop the perpetuating cycle of oxidation of cell membranes (Gonzalez Flecha et al., 1991; Serbinova et al., 1991). As a fat-soluble vitamin, it has the ability to protect tissues with high lipid content (Lee and Han, 2018). Due to the large amount of polyunsaturated fatty acids in the brain, this organ is at high risk for oxidative damage (Cobley et al., 2018), making vitamin E essential for brain health. Animal seizure models have demonstrated that pharmacological doses of vitamin E administered after a kainate-induced seizure, can almost completely restore function of glutamine synthase, the enzyme responsible for converting excitatory glutamate, into non-toxic glutamine, for transport between astrocytes and neurons (Ambrogini et al., 2019). There is some *in vitro* data which suggests that vitamin E may be able to reduce activity of the ionotropic AMPA, kainate, and NMDA glutamate receptors, in addition to protecting mitochondrial membrane potential through its antioxidant function (Abedi et al., 2017). Additionally, there is *in vitro* and *in vivo* evidence that vitamin E can reduce neuroinflammatory responses mediated by microglial cells (Li et al., 2001; Gonzalez-Perez et al., 2002; Godbout et al., 2004**;** Stolzing et al., 2006**;** Annahazi et al., 2007), including studies showing that treatment with alpha-tocopherol lowered hippocampal levels of IL-1β and TNF-α in an animal model of epilepsy (Ambrogini et al., 2019).

As mentioned earlier, antioxidants work together to maintain themselves in their reduced form. Vitamin E can be reduced to its active form by vitamin C and the ascorbate form of vitamin C can be regenerated by thiols such as glutathione (Packer et al., 2001). Thus, these three antioxidants have synergistic action to protect against oxidative stress in the nervous system.

## Glutathione

Glutathione (GSH) is an extremely important endogenous antioxidant that is a tripeptide formed from the combination of glutamate, cysteine, and glycine (Huang et al., 1993). The availability of cysteine is the rate limiting factor in its production (Huang et al., 1993). Approximately 10–15% of intracellular GSH can be found in the mitochondria, and reduced GSH levels have been associated with mitochondrial dysfunction, including low production of ATP (Enns et al., 2014). In order for GSH to be produced and maintained in its reduced state, a few vitamins are needed, including vitamin D and riboflavin. Vitamin D plays an important supportive role in antioxidant function through its modulation of gene transcription for antioxidant enzyme systems like glutathione peroxidase (Bhat and Ismail, 2015; Tohari et al., 2016; Masjedi et al., 2020). Vitamin D can also upregulate production of the enzymes needed for GSH synthesis and reduction, thereby upregulating its production and antioxidant activity (Jain and Micinski, 2013). Of important note, vitamin D may also be able to reduce excitotoxicity through its action on L-type calcium channels which are thought to modulate NMDA receptors (Brewer et al., 2001), to reduce microglial activation in an LPS model of inflammation (Hur et al., 2014), and to increase release of the anti-inflammatory cytokine, IL-10 (Boontanrart et al., 2016), making it of even greater importance. In addition to vitamin D, riboflavin is also necessary for glutathione function as it serves as a cofactor for glutathione reductase, which is the enzyme responsible for maintaining GSH in its reduced form (Beutler, 1969). Similarly, vitamins B6, folate, and B12 work together in one carbon metabolism (Selhub, 2002) to provide cysteine for the production of glutathione (Dalto and Matte, 2017), and this also allows glutamate to be used in the production of glutathione, thereby limiting glutamate availability for excitotoxicity (Wu et al., 2004).

## Riboflavin (Vitamin B2)

In addition to vitamin C, vitamin E, and glutathione, riboflavin (vitamin B2) also appears to play an important role in affecting redox activity *in vivo*, countering excitotoxicity, and protecting against neuroinflammation. In addition to the supportive role of riboflavin for glutathione reductase mentioned above (Beutler, 1969), riboflavin also has the ability to affect ROS in multiple ways. First, riboflavin has been shown to have its own antioxidant activity, where the reduced form of riboflavin was able to deactivate lipid peroxides (Ashoori and Saedisomeolia, 2014). Other research has demonstrated riboflavin’s activity against mutagen produced free radicals (Durusoy et al., 2002). Second, early data suggests that riboflavin may also have the ability to affect superoxide dismutase (SOD) and catalase (Huang et al., 2010). Thus, it appears that riboflavin may be a key micronutrient for supporting antioxidant activity, though more research in humans is needed.

Riboflavin also protects against excitotoxicity in multiple ways. First, riboflavin is required for the production of the active form of vitamin B6, pyridoxal phosphate (PLP; Adelekan et al., 1987), which is necessary for the formation of many neurotransmitters in the nervous system (Spinneker et al., 2007). One of the neurotransmitter pathways where PLP is active, is as a cofactor for glutamate decarboxylase, which converts glutamate into gamma aminobutyric acid (GABA). GABA is the major inhibitory neurotransmitter in the nervous system and ideally needs to be in balance with glutamate for optimal neuronal functioning (Petroff, 2002). A deficiency in riboflavin can lead to reduced production of PLP [essentially a vitamin B6 deficiency (Madigan et al., 1998)], preventing it from serving as a cofactor in the production of GABA (Spinneker et al., 2007); meaning that riboflavin deficiency can cause increased glutamate and deceased GABA levels, thereby contributing to excitotoxicity. Riboflavin and PLP are also essential cofactors in the kynurenine pathway (Marashly and Bohlega, 2017), where adequate intake of these vitamins has been associated with production of kynurenic acid (Theofylaktopoulou et al., 2014), rather than quinolinic acid, which acts as an agonist to the NMDA receptor, thereby potentiating excitotoxicity (Zinger et al., 2011; Curto et al., 2015). Riboflavin may also be able to directly affect excitotoxicity by inhibiting the exocytosis of glutamate vesicles in presynaptic neurons (Wang et al., 2008). Finally, riboflavin (along with PLP, folate, and vitamin B12) also has the ability to help protect against homocysteine build-up (Rozycka et al., 2013). Homocysteine has been shown to be neurotoxic via its ability to act as an agonist at the NMDA receptor (Deep et al., 2019), making riboflavin’s ability to reduce homocysteine very valuable.

Riboflavin additionally has the ability to protect against neuroinflammation. First, riboflavin was shown to effectively reduce TNF-α, IL-1β, and nitric oxide (NO) in a staphylococcus infection model (Dey and Bishayi, 2016). Riboflavin also plays an indirect role for opposing inflammation through its effects on vitamin D metabolism. Multiple enzymes in the biosynthetic pathway of vitamin D are dependent on riboflavin for their action (Pinto and Cooper, 2014). Animal models have been able to induce vitamin D deficiency from riboflavin deficiency due to this effect on the internal synthesis of vitamin D (Pinto and Cooper, 2014). Since vitamin D has anti-inflammatory effects (as detailed above), riboflavin deficiency may inhibit this protective function of vitamin D.

### Other Micronutrients of Interest

In addition to the above micronutrients, there are a few others which also affect two aspects of the neurotoxic triad. In addition to vitamin B6’s role in glutathione production, it also acts as cofactor in the conversion of glutamate to GABA (Martin, 1987), with deficiency leading to increased excitation and decreased inhibition, thereby potentiating effects of excitotoxicity (Baumeister et al., 1994). Moreover, it has the ability, along with folate and vitamin B12, to limit the production of homocysteine (Zaric et al., 2019), which can act as a neurotoxic agonist of the NMDA receptor (Deep et al., 2019). There is also some data to suggest that vitamin B6 and B12 deficiency may be associated with increased inflammation (Al-Daghri et al., 2016; Ueland et al., 2017). Finally, the mineral magnesium serves as a block of the NMDA receptor, helping to protect against excitotoxicity (Nikolaev et al., 2012), and also appears to reduce microglial activation and downstream production of inflammatory cytokines (Maier et al., 2021).

## Potential for Deficiencies

Food is very impactful for human health in general, and the dietary components reviewed above are great examples of how important food may be for optimizing health of the nervous system and for treating neurological conditions which are characterized by the neurotoxic triad. Food sources for each of the micronutrients is included in Table 1 for easy reference. Deficiencies in many of these micronutrients are common. Inadequate consumption of vitamin C is thought to be common in the United States (Hampl et al., 2004). Similarly, current estimates suggest that the majority of the population may not be receiving adequate quantities of vitamin E (McBurney et al., 2015). Vitamin D deficiency is also very common in populations living at more extreme latitudes due to reduced exposure to UV light, and is further compounded in those who do not eat fish frequently (Holick, 2004) and who do not compensate in some manner such as taking cod liver oil during the colder months. Finally, riboflavin has also been shown to be under-consumed, with estimates suggesting that 10–15% of the global population may be deficient, and over 50% of non-elderly British adults were estimated to have borderline deficiency based on glutathione reductase activation in erythrocytes (Kennedy, 2016). Deficiency of vitamin B12 is common in vegetarians and vegans, as well as populations suffering from protein malnutrition (Stabler and Allen, 2004), and 45% of Americans are thought to be deficient in magnesium (Workinger et al., 2018). Therefore, increased research attention on the effects of these micronutrients is warranted.

## Conclusion

The neurotoxic triad is characterized by excitotoxicity, oxidative stress, and neuroinflammation, with each aspect of the triad being able to perpetuate the others inside of the nervous system. It has been suggested in the literature that to adequately address oxidative stress as a contributor to neurodegeneration, that we must also simultaneously address excitotoxicity (Li et al., 2013), but dietary micronutrients may offer an even better solution by reducing all three aspects of the neurotoxic triad, including neuroinflammation. Vitamin C, vitamin E, vitamin D, and riboflavin are key dietary antioxidants which simultaneously protect against excitotoxicity, oxidative stress, and neuroinflammation. Similarly, glutathione also appears to directly affect all three aspects of the neurotoxic triad. Future dietary research should examine how increased intake of these micronutrients, along with other nutrients like vitamins B6 and B12, and magnesium, may be protective against excitotoxicity, oxidative stress, and neuroinflammation.

## Data Availability Statement

The original contributions presented in the study are included in the article/supplementary material, further inquiries can be directed to the corresponding author.

## Author Contributions

KH was responsible for the design, research, writing, and final content of this manuscript.

## Conflict of Interest

The author declares that the research was conducted in the absence of any commercial or financial relationships that could be construed as a potential conflict of interest.

## Publisher’s Note

All claims expressed in this article are solely those of the authors and do not necessarily represent those of their affiliated organizations, or those of the publisher, the editors and the reviewers. Any product that may be evaluated in this article, or claim that may be made by its manufacturer, is not guaranteed or endorsed by the publisher.
